# Supplementary material for: Combined Application of Myo-Inositol and Corn Steep Liquor from Agricultural Waste Alleviate Salt Stress in Brassica rapa
Source: Plants (Basel). 2023 Dec 8;12(24):4110. doi: 10.3390/plants12244110 (PMC10748129; doi:10.3390/plants12244110)
Supplement: Supplementary file 1 [file plants-12-04110-s001.zip › plants-2755189-supplementary.pdf]

plants

MDPI

Figure S1

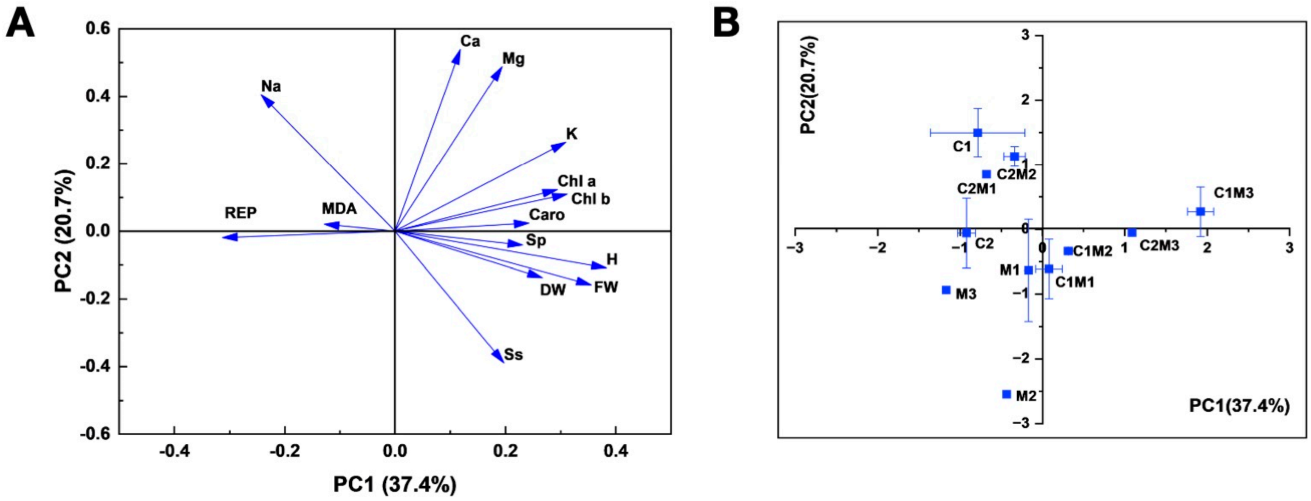

Figure S1: Analysis of the effects of CSL, MI, and their combination on the growth and physiological traits of cabbage under salt stress conditions (excluding the control combination alone salt treatment). (A,B) Effect of MI, CSL, and their complexes on principal component analysis (PCA) of cabbage indicators under salt stress. The direction and length of the blue arrows indicate the correlation. FW: fresh weight; DW: dry weight; H: plant height; Chl a: chlorophyll a; Chl b: chlorophyll b; Caro: carotenoid; Pro: proline; Sp: soluble protein; Ss: soluble sugar; REP: electrolyte leakage; MDA: malondialdehyde.
